# DNA Diagnostics for Schistosomiasis Control

**DOI:** 10.3390/tropicalmed3030081

**Published:** 2018-08-01

**Authors:** Kosala G. Weerakoon, Catherine A. Gordon, Donald P. McManus

**Affiliations:** 1 Molecular Parasitology Laboratory, Infectious Diseases Division, QIMR Berghofer Medical Research Institute, Brisbane 4006, Australia; Catherine.Gordon@qimrberghofer.edu.au; 2 School of Public Health, University of Queensland, Brisbane 4006, Australia; 3 Department of Parasitology, Faculty of Medicine and Allied Sciences, Rajarata University of Sri Lanka, Saliyapura 50008, Sri Lanka

**Keywords:** schistosomiasis, diagnosis, control and elimination, DNA, polymerase chain reaction

## Abstract

Despite extensive efforts over the last few decades, the global disease burden of schistosomiasis still remains unacceptably high. This could partly be attributed to the lack of accurate diagnostic tools for detecting human and animal schistosome infections in endemic areas. In low transmission and low prevalence areas where schistosomiasis elimination is targeted, case detection requires a test that is highly sensitive. Diagnostic tests with low sensitivity will miss individuals with low infection intensity and these will continue to contribute to transmission, thereby interfering with the efficacy of the control measures operating. Of the many diagnostic approaches undertaken to date, the detection of schistosome DNA using DNA amplification techniques including polymerase chain reaction (PCR) provide valuable adjuncts to more conventional microscopic and serological methods, due their accuracy, high sensitivity, and the capacity to detect early pre-patent infections. Furthermore, DNA-based methods represent important screening tools, particularly in those endemic areas with ongoing control where infection prevalence and intensity have been reduced to very low levels. Here we review the role of DNA diagnostics in the path towards the control and elimination of schistosomiasis.

## 1. Introduction

The public health and socioeconomic impact of schistosomiasis is such that, to date, over 230 million people have acquired the disease, including many children, mainly in the tropics and subtropics. Further, this chronic debilitating disease leads to around 11,500 deaths a year and it is responsible for the loss of over 3.5 million DALYs, the majority (more than 80%) from sub-Saharan Africa [1,2]. The major schistosome species that cause infection in humans include *S. haematobium*, the agent of urinary schistosomiasis, and *S. mansoni*, *S. japonicum*, *S. mekongi*, *S. intercalatum* and *S. guineensis*, which cause intestinal schistosomiasis. These blood-feeding flukes are responsible for substantial long-term clinical complications with multiple organ involvement including the liver, intestine, and urinary bladder. Infective cercariae in fresh water sources penetrate the host skin and enter the blood circulation as schistosomules and inhabit mesenteric or vesical (intestinal and urinary schistosomiasis respectively) venous plexuses after pulmonary and hepatic migrations. Mature female worms lay eggs in these sites, and eggs then penetrate the intestinal walls (in intestinal schistosomiasis) to be excreted in stool or penetrate the bladder wall (in urinary schistosomiasis) to be excreted in urine while some of the eggs migrate towards ectopic sites such as the liver and other organs, leading to chronic inflammation and fibrosis. The eggs released to the environment hatch in fresh water sources releasing miracidia that penetrate specific snail hosts within which they undergo asexual reproduction and become cercariae to continue the life cycle. 

Successful disease prevention and elimination programs for schistosomiasis involve the implementation of intensive intervention and efficient monitoring measures, with different countries having their own modified approaches tailored to the sociocultural and economic situations prevailing [3,4,5]. For example, in China the number of human schistosomiasis cases was reduced by 90% over the decade from 2004 through human case detection and treatment, health education and snail control [6]. Additionally, China has had a strong political will for many decades to eliminate schistosomiasis, since control options were first instigated by Chairman Mao in 1956, who made its elimination a national health priority [7]. In general, accurate community diagnosis of the infection and continued surveillance is helpful in the control of transmission of schistosomiasis, while prompt treatment following early detection can minimize the associated morbidity and mortality [8,9]. With continuing multiple prevention and control efforts, the prevalence and intensity of schistosomiasis in many endemic regions have gone down, so that in many infected individuals, the disease may go undetected with commonly-used conventional diagnostic tools such as the Kato-Katz fecal smear (KK) test or urine egg filtration methods, due to their low sensitivity [10,11,12,13]. As a result, a schistosomiasis-endemic area may appear to be free of the disease infection whereas in reality transmission continues and may even spread to other communities, thereby increasing the time for control and eventual elimination. A recent World Health Organization (WHO) expert committee report [12] highlighted the significance of a One Health approach focusing on preventive chemotherapy, improvement of water, sanitation and hygiene (WASH), health promotion, snail control, and detection and treatment of animal reservoirs for the sustained control and elimination of Asian schistosomiasis [12]. This further emphasizes the importance and essential need for accurate diagnostics, if the target goals of transmission interruption by 2025 and elimination of transmission by 2030 are to be achieved.

## 2. A General Overview of Diagnostics for Schistosomiasis

**Figure 1:**
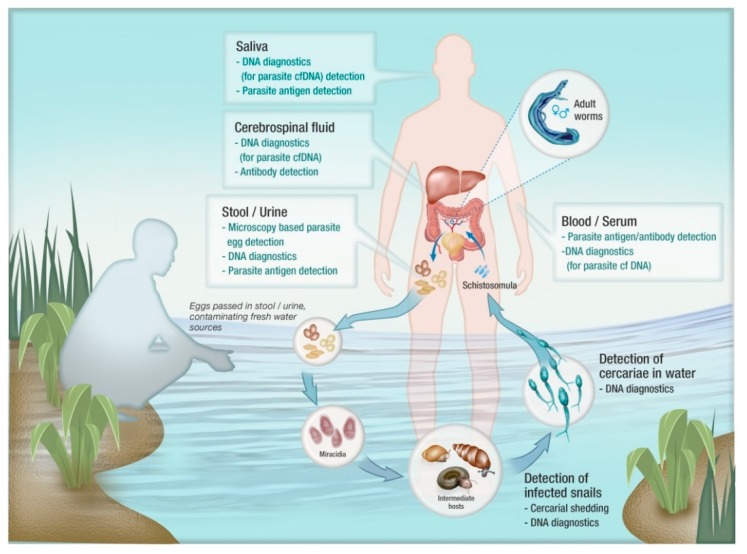
Applicability of diagnostic tools for the detection of different lifecycle stages of schistosomes.

Procedures that have been commonly applied in schistosomiasis diagnosis include conventional microscopy-based tests, different antibody-based serological assays, parasite antigen detection assays and DNA detection methods including polymerase chain reaction (PCR)-based procedures (Figure 1). As considered earlier, the KK and microscopy-based egg detection in urine have the major drawbacks of low sensitivity and can be labor intensive [14,15,16,17]. Antibody detection assays also have low diagnostic accuracy, particularly in terms of test specificity, as well as being unable to distinguish between past and current infections [16]. Recent improvements in circulating parasite antigen detection assays have resulted in relatively higher accuracy in comparison with microscopic and antibody detection methods but frequent fluctuations in assay replicates have suggested the need for multiple testing to improve diagnostic accuracy [18,19].

Furthermore, the circulating antigen (circulating anodic antigen (CAA) and circulating cathodic antigen (CCA))-based assays are not currently applicable for all schistosome species. The CCA-based assay is used as a point of care test to diagnose *S. mansoni* but does not work for *S. haematobium* [20]. CAA-based lateral flow assays combined with up-converting phosphor reporter technology, work for both *S. mansoni* and *S. haematobium*. However, they have not as yet proven as effective with other schistosome species [20,21,22]. As a result, DNA, especially PCR-based parasite DNA detection assays, have stimulated much interest as alternative options due to their proven diagnostic accuracy, higher sensitivity, and wider range of applicability, including the ability to detect early pre-patent infections. Here we review the different DNA detection methods that have been employed in the diagnosis of human and animal schistosomiasis, discuss their application in control programs and consider their value in surveillance leading to elimination goals.

## 3. DNA-Based Diagnostics for Schistosomiasis

Technological advances and the substantial genomic data now available for schistosomes [23,24,25] have opened up new avenues for the development of novel diagnostics as well as identifying new therapeutic and vaccine targets. Recent advances in DNA amplification assays include the application of real-time quantitative PCR (qPCR) and droplet digital PCR (ddPCR) for the detection of circulating cell-free parasite DNA using different clinical samples and the development of isothermal amplification assays such as loop-mediated isothermal amplification (LAMP) and recombinase polymerase amplification (RPA) techniques [26,27,28,29]. Measures of accuracy including diagnostic sensitivity and specificity of DNA detection assays vary depending on the type of the assay, target gene sequence used, as well as the type of sample tested [30]. Some of the key advantages and limitations of these different DNA detection assays along with the relative costs involved are summarized in Table 1. One of the key factors in the development of a highly sensitive DNA amplification assay is the selection of a specific amplification target sequence with numerous copies that is available in abundance within a single parasite cell; such sequences include both nuclear and mitochondrial genes [27,30,31,32,33]. In addition to being highly abundant, a target gene sequence for diagnostic DNA amplification needs to be highly specific for the targeted species so that the resulting assay is highly sensitive and specific. The *SjR2* retrotransposon [34,35,36] and the *nad1* mitochondrial gene [37,38] are two target sequences that have been commonly used in the diagnosis of *S. japonicum* infection. Similarly, the *SM1-7* tandem repeat sequence [39] and 18S rDNA are two targets used in the diagnosis of *S. mansoni* while mitochondrial *cox1* [40] and the *Dra1* repeat sequence [41] have been utilized for *S. haematobium* detection. 

### 3.1. Sample Preservation and DNA Isolation for DNA Amplification Assays

DNA extraction is a key procedure but it can be a methodological bottleneck in PCR-based diagnostic assays since the yield and quality of DNA directly affects the outcome of the amplification procedure; it is also often the most expensive part of DNA-based diagnosis, particularly when using commercially-available extraction kits [55,56]. Furthermore, sample collection and preservation techniques have a significant influence on DNA extraction and amplification outcomes. DNA extraction from fresh clinical samples is not feasible in the field and generally needs to be performed in a central laboratory. Hence appropriate measures are required to preserve samples until DNA extraction can be undertaken. These methods can vary depending on the type of clinical sample involved [56,57]. For example, common methods used in plasma sample preservation and storage include addition of K3EDTA at the time of blood sampling, and freezing [57]. K3EDTA is preferred over heparin as it stabilizes DNA and, unlike heparin, does not inhibit downstream amplification reactions [57]. Similarly, fecal samples for parasite DNA extraction can be readily preserved by immediate freezing, storage with alcohol, addition of commercial solutions such as RNA later and PAXgene, and preservation on Whatman FTA cards [58]. However, these preservation/storage reagents need to be carefully removed prior to DNA extraction since they can interfere with DNA yield as well as downstream assay procedures [58]. 

Major aims of DNA isolation for PCR include the removal of PCR inhibitors and nucleases, and maximizing DNA recovery and quality of DNA. In the diagnosis of schistosomiasis, stool and urine are the most commonly used clinical samples for DNA isolation; other bio-fluids (serum, saliva, and cerebrospinal fluid) are also used, particularly for cell-free DNA (cfDNA) detections assays. Conventional DNA extraction methods include precipitation techniques with phenol-chloroform and ethanol or isopropanol. However, the feasibility of applying these methods has in the past been affected by the potential for direct exposure of hazardous chemicals to operators and the significant time involved. However, the availability of commercial DNA extraction kits has markedly reduced these limitations and improved the quality and yield of isolated DNA [59,60]. The protocols involve techniques such as spin column-based purification and magnetic bead DNA separation. Furthermore, these kits have been developed for use on different clinical sample types to overcome the unique challenges related to each. Moreover, recent advances in DNA purification technology have resulted in high-throughput automated platforms minimizing the time spent, the labor involved and manual handling, making the process convenient, efficient and accurate [61,62,63]. However, as is common to most high-tech applications these automated techniques involve costly equipment, and are currently not suitable for field application in disease-endemic, resource-poor settings. 

Most DNA amplification methods generally need to be performed in well-equipped centralized facilities that are generally located distant from the clinical specimen collection site. Therefore, under these circumstances it is important that feasible and rapid sample preservation methods are available prior to DNA extraction and amplification. Potential solutions to these issues include the preservation of clinical specimens such as fingerprick blood, urine, or stool on filter papers as dried spots for convenient transportation to a central laboratory for DNA extraction and amplification, or for direct PCR [45,64,65,66].

### 3.2. Conventional and Quantitative Real Time PCR

Of nucleic acid amplification tests, conventional PCR (cPCR) was developed first. A key advantage of the technique is the ability to observe the amplification products corresponding to appropriate base pair size that can be conveniently used in specific genomic detection through sequencing. cPCR has been applied in different surveys including the evaluation of therapeutic responses in schistosomiasis [16,67]. Another important aspect of cPCR is the feasibility to develop a multiplex assay to detect multiple infections within a single clinical sample [68]. Multiplexing in cPCR requires differing target amplicon sizes to be distinctively identified in gel electrophoresis. Specific target gene segment detection in stool or urine samples using cPCR has been widely applied in the diagnosis of schistosomiasis [42,43,44]. The method is highly sensitive compared with conventional microscopy methods, particularly with low intensity infections [33,43,69,70]. The accuracy of cPCR assays has been improved with the combination of other techniques, such as PCR-ELISA analysis [71] and restriction fragment length polymorphism analysis of PCR amplified fragments (PCR-RFLP) for the diagnosis of schistosome infections. The PCR-RFLP technique involves restriction endonucleases digestion of the PCR amplicon, giving rise to different electrophoretic patterns thereby providing a method for simultaneous analysis of multiple species/strains [72]. This technique has been used in characterization of snails as well as in the detection of schistosomiasis and other helminth infections [72,73,74,75].

Nested PCR (nPCR) is a more sensitive and a specific approach than cPCR, and has been successfully applied in different instances in the diagnosis of schistosomiasis [46,76]. However, the procedure includes duplication of cPCR for the initial amplification of a larger gene fragment and then another sequence within the initial fragment, which involves more labor and a higher cost. 

qPCR has been widely applied in the detection of human schistosome infections of different species including large-scale epidemiological surveys and monitoring of the therapeutic response. The qPCR technique is generally more sensitive than cPCR and, importantly, can provide a measurement of infection intensity. Additionally, the procedures involved with qPCR are streamlined compared with cPCR which needs an additional electrophoresis step to detect PCR end-products [27,77,78]. Another important characteristic of qPCR is that, like cPCR, it has the ability to utilise multiplex assays to detect multiple infections within a single clinical sample using specific DNA probes. Generally, however, qPCR assays are preferred over cPCR in the development of multiplex assays, having the additional advantage of improved specificity with the use of probes and convenience in high-throughput applications. Moreover, recent studies have demonstrated the ability to detect a very high spectrum of parasites in a single sample (multi-parallel PCR) further improving multiplex qPCR assays [79,80,81,82]. Multiplexing capability has clear practical significance largely in terms of cost-effective application in epidemiological studies and for monitoring of schistosomiasis control programs, particularly in co-endemic settings, an example being the detection of *S. mansoni* and *S. haematobium* in human fecal or urine samples in areas where both species are present [27,83]. 

### 3.3. Loop-Mediated Isothermal Amplification (LAMP)

The LAMP technique is a relatively simple, cost-effective and rapid DNA detection approach compared with the commonly-used PCR-based assays and is more field-friendly. Application of the assay does not require specific equipment such as a thermocycler, electrophoresis apparatus or gel documentation units [84]; hence it is simple, and applicable in resource-poor settings once optimized. Moreover, the use of specific inner and outer primer sets makes the assay highly specific to the intended target sequence, combined with high sensitivity [85,86]. However, the initial optimization process is complicated and time consuming with the use of multiple primers. Furthermore, it is known that the LAMP technique, being highly sensitive, is highly vulnerable to carryover contamination of LAMP products from previous reactions, which can be re-amplified leading to false-positive results [87,88].

LAMP assays have demonstrated high sensitivity in the detection of *S. mansoni* and *S. haematobium* infections in co-endemic areas using urine samples, indicating the possible use of the technique as a point of care (POC) diagnostic [65]. Furthermore, a LAMP assay has been used in the sensitive detection of early pre-patent schistosome infection in an animal model [89]. In a recent field survey to detect *S. mansoni* infection in a low-transmission area, a LAMP assay was successfully applied in both snail and human stool samples and the study emphasized the potential application of this molecular approach for the identification of transmission foci and for building risk maps in support of control programs [48]. Furthermore, the LAMP method was successfully used recently in China for surveillance, including in snail surveys, indicating its usefulness, and applicability as a rapid screening and environmental risk assessment tool to identify areas suitable for targeted intervention [12]. 

Recent research has raised the possibility of developing multiplex LAMP assays, a concept that could be adapted for the diagnosis of multiple parasitic species, including different schistosomes, in infected individuals. Multiplex LAMP procedures incorporate an additional endpoint readout option to discriminate between amplified products, such as melting curve analysis to test for different melting temperatures or detection of distinctive gel-electrophoretic banding patterns reflecting different species characters [90,91]. The LAMP technique could provide an effective method, applicable in resource-poor endemic communities, to diagnose co-infections of *S. mansoni* and *S. haematobium*, multiple co-infections of soil-transmitted helminths (STH), or co-infections of intestinal protozoa and schistosomes, as achieved using qPCR [27,92,93,94].

### 3.4. Recombinase Polymerase Amplification (RPA)

The RPA technique is another isothermal amplification technique usually conducted under lower temperatures (around 40 °C). In RPA, DNA sequences are amplified with the use of DNA polymerase, DNA binding proteins and recombinase; primer binding to the template DNA is facilitated by nucleoprotein complexes made of recombinase proteins and oligonucleotide primers [49,95]. Similar to LAMP, the application of the RPA technique is straight forward and applicable in resource-poor settings since it does not require specific equipment such as a thermocycler, electrophoresis apparatus or gel documentations units. This novel technique has now been integrated with a chip and lateral flow devices making it a convenient portable application as a point of care diagnostic tool [95,96]. RPA has been applied in the diagnosis of both intestinal and urinary schistosomiasis, including its use in field evaluation, and has been shown to be superior to microscopy and serology in terms of convenience, detection time and diagnostic sensitivity [49,50,51]. However, the technique does have some practical limitations such as the need for transferring amplified products to the detection device, which can lead to potential nucleic acid contamination resulting in false positives [50,96].

### 3.5. Droplet Digital PCR (ddPCR)

Due to a recent advance in PCR technology, ddPCR is proving to be more sensitive and precise compared with qPCR [97,98,99]. ddPCR has been successfully used in the detection of cfDNA and in the diagnosis of infections and other clinical conditions, including cancer [53,97,100,101,102,103,104]. Moreover, it was recently applied in the diagnosis of *S. japonicum* in both an animal model and in diverse human clinical samples, and was able to quantify intensity of infection in terms of direct target gene copy number [35,53]. The technique can also be used for multiple target testing, thereby providing an effective diagnostic approach for detecting multiple parasites in an infected individual [105,106].

### 3.6. Direct PCR

The need for a prior DNA isolation step is a major limitation in routine PCR analysis, resulting in the requirement for additional resources and costs, delays in obtaining a result, and added complexity. Attempts have been made to overcome this constraint by optimizing PCR protocols so that clinical specimens can be added directly into the PCR reaction. However, the initial development and optimization of a ‘direct PCR’ assay has multiple challenges, particularly the potential negative effect of PCR inhibitors present in clinical samples. Application of modified, inhibitor-resistant polymerases and inclusion of additional reagents such as chelating agents and protease inhibitors are some strategies used to overcome these effects [107,108]. Direct application of the PCR technique has been undertaken in clinical diagnosis, including the identification of parasitic infections, with the use of conveniently preserved clinical samples such as dry blood spots [109,110]. This technique has been tested for applicability in schistosomiasis diagnosis using animal models [54], and its further improvement and evaluation would be a helpful advance for undertaking molecular diagnostics directly under field conditions rather than in a central laboratory, far from the disease-endemic community.

### 3.7. Parasite cfDNA Detection in Clinical Samples

Application of the PCR technique was mostly restricted to schistosome egg DNA detection until the recent development of parasite cfDNA detection methods in different clinical samples. Compared with the DNA originating from schistosome eggs in stool or urine samples, cfDNA is generally homogenously distributed in plasma and other bodily fluids, which potentially eliminates one of the major sampling problems associated with copro PCR or urine PCR, where eggs are the primary source of DNA [16,33,35,53,111,112]. Parasite cfDNA is released from schistosome stages (schistosomula, adult worms and eggs) within the mammalian host, and could possibly be the result of dead or decaying parasites within the circulation and tissues, active shedding from the parasite or from disintegrating inactive eggs [29,113,114]. Infections with all the three major human schistosomes have been diagnosed using DNA amplification-based cfDNA detection assays, and parasite cfDNA can be detected in host serum in early prepatent schistosomiasis [115,116,117]. In addition to serum/plasma, parasite cfDNA can be excreted in bodily fluids such as urine, saliva, and cerebrospinal fluid, and can be effectively quantified using qPCR and ddPCR assays [33,53,112,118,119]. 

As it is likely that the amount of parasite cfDNA in a given clinical sample will be low compared with one containing parasite eggs, a higher level of detection sensitivityis imperative. Of the different PCR-based DNA detection methods available, ddPCR and qPCR are optimal for this purpose [35,52,53]. Recent studies have demonstrated the successful amplification of the *SjR2* retrotransposon gene and *nad1* mitochondrial gene for the detection of both early pre-patent and late *S. japonicum* infection [35,53]. Furthermore, detection of cfDNA using ddPCR and qPCR has proven to be effective in individual case detection, in large scale field application and for monitoring therapeutic responses [111,118,120]. 

## 4. Applications of DNA Diagnostics for Schistosomiasis

### 4.1. Individual Case Detection

Early confirmation of the diagnosis of acute schistosome infection is imperative for early intervention and to achieve a good prognosis for the patient with minimum complications. Acute schistosomiasis cases include returned travelers, immigrants, and refugees [121,122], where patients present to health care facilities with early clinical manifestations such as cercarial dermatitis. These patients need to be carefully clinically evaluated and investigated but most of the commonly applied diagnostics are unable to detect these pre-patent schistosome infections. Schistosome cfDNA detection is an ideal option to diagnose these cases either using serum or non-invasive clinical samples like urine [114]. Furthermore, cfDNA detection is helpful in situations such as neuroschistosomiasis, where parasite DNA can be detected in host cerebrospinal fluid [46,120].

### 4.2. Diagnosis of Zoonotic Schistosomiasis in Animal Reservoirs

Accurate diagnosis of *S. japonicum*-infected mammalian reservoirs is key to achievingthe elimination of zoonotic schistosomiasis in China and the Philippines [42,123,124]. Similar to the diagnosis of human cases, insensitive conventional microscopy-based diagnostic procedures often do not detect infected animals that continue to contribute to schistosomiasis transmission. DNA amplification-based methods have now been shown to be highly effective in the diagnosis of animal reservoirs of Asian schistosomiasis. Recent surveys undertaken on carabao in the Philippines disclosed a substantially higher prevalence of schistosomiasis using qPCR on fecal samples, compared with copro-microscopic diagnosis [42,125]. Moreover, a recently-developed nPCR assay has also shown potential for field application in the sensitive diagnosis of early cases of schistosomiasis in domestic animals, using serum samples [45].

### 4.3. Detection of Infected Snail Hosts

*Bulinus* spp., *Biomphalaria* spp., and *Oncomelania* spp. act as the intermediate hosts of *S. haematobium*, *S. mansoni* and *S. japonicum* respectively. Detection of infected snail hosts—xeno-monitoring—is a pivotal indicator of an existence of schistosomiasis in a particular area, and the potential for transmission. Furthermore, xeno-monitoring is important in identifying infection risk areas to guide surveillance and necessary interventions, and represents a critical measure for achieving schistosomiasis elimination goals [126,127,128]. The example of Japan is one of the best to show the importance of snail control in schistosomiasis elimination, where the main control strategy was to target susceptible snail colonies using chemical molluscicides and environmental modifications [129].

Commonly used techniques in xeno-monitoring include cercarial shedding with light exposure, microscopic detection of sporocysts and cercariae in crushed snails. These traditional methods have detection limitations particularly in situations where there is a low parasite burden, or where there is aborted development of sporocysts [130]. Moreover, the labor-intensive nature of these procedures, including the collection and handling of snails and the associated costs, are both major disadvantages [131]. 

Molecular tools are now being widely applied in the detection of schistosome-infected snail hosts, providing promising results in support of control and elimination efforts particularly with large scale screening programs [132]. One early study described the detection of *S. haematobium* in *Bulinus truncatus* snails using cPCR targeting the *DraI* repeat sequence [128]. As well as demonstrating high sensitivity, the study highlighted the cost-effective application of the assay through the grouping and pooling of snails in the analysis. Moreover, PCR and LAMP assays were used in recent studies to detect *S. mansoni* DNA in *Biomphalaria* snails [133,134]. One of the studies showed that the LAMP assay could detect one infected snail within a pool of a thousand uninfected snails [133]. Hence this approach can provide an important low cost, rapid and highly sensitive tool for the monitoring of infected snails to provide important information required before appropriate control measures are undertaken [133]. Similar efforts have also been successful in the detection of *S. japonicum*-infected *Oncomelania* snails using LAMP-based DNA detection [135]. The application of multiplex qPCR assays have the additional advantage of identifying both snail and infecting schistosome species, another helpful consideration for successful schistosome and snail control programs [136]. Furthermore, molecular methods are of considerable help in developing transmission risk maps prior to the instigation of control efforts [48,135].

### 4.4. Surveillance of Environmental Sources

In addition to xeno-monitoring, the other important surveillance measure to determine the existence of environmental contamination with schistosomes is the detection of miracidia and cercariae in water sources. Evaluating the presence of cercariae is an important factor in detecting infection transmission sites. Commonly used conventional microscopic methods lack sensitivity and are highly labor-intensive [137,138]. Testing for the presence of cercariae is helpful in determining their diurnal variation, seasonal patterns, and spatial distribution. PCR-based molecular tools are now being increasingly applied to this area of surveillance [131,139,140]. qPCR has been successfully used in quantitative detection of *S. japonicum* cercariae in water samples, showing potential for the rapid and high throughput analysis of environmental samples and its application in the field [140,141]. 

### 4.5. Assessment of Progress of Control Measures

One of the key elements of sustained efforts on control and elimination measures is the frequent monitoring and surveillance of the effects/progress of the strategies already implemented. While effective control measures need to be continued with high priority, identification of less effective interventions are important in early application of appropriate modifications to improve the effect on control and elimination efforts. Application of highly sensitive and accurate diagnostics is imperative for this purpose since the ongoing control measures lead to lowered prevalence of infection and lowered infection intensity in the community [6,131,142,143]. Further, these assays also need to have the capability to perform in a short time period, with a minimum requirement of equipment and expertise and they have to be cost-effective [144]. The equipment, reagents, and setup required for conventional diagnostics, particularly parasitological methods, are relatively inexpensive, but they can be laborious especially as they need to be repeated multiple times to reach a certain diagnostic accuracy, which can also affect the costs involved. Both molecular and serological diagnostics performed in specific laboratories are more expensive, particularly with the requirements of costly equipment and reagents, specific maintenance requirements, and the need of trained personnel to carry out the tests. Field/community applicable POC immunodiagnostics are simple to use and require only a minimum amount of labor and equipment, but may involve a higher production cost [145,146]. As a result, despite being highly sensitive and accurate, DNA diagnostics cannot currently completely replace the conventional diagnostic methods for community, field, and environmental surveillance. However, the combined application of different techniques is a reasonable approach whereby, for example, an initial diagnosis is undertaken with traditional methods followed by further screening of any test-negatives with molecular tools to capture any missed infections. 

## 5. Challenges and the Way Forward

While there has been substantial focus on mass drug administration (MDA) as a measure to control and eliminate schistosomiasis, efforts to establish accurate, field-deployable novel diagnostics, particularly in resource-poor endemic settings, have been comparatively limited. Microscopy-basedprocedures are recognized as being imprecise, making it important that more sensitive diagnostic tools are developed and deployed. Parasite DNA amplification-based molecular tools are showing encouraging promise towards reaching the level of sensitivity and specificity required to monitor the effectiveness of control programs that will lead to the elimination of schistosomiasis. These molecular assays have a wide range of applications including human case detection, detection of infection in animal reservoir hosts and snails, and in environmental surveillance, which are essential requirements to achieve elimination targets.

Despite the fact that highly sensitive and accurate, ddPCR technology is not yet at the level where it can be used routinely in the field. This is likely in the near future as it can be readily applied for the diagnosis of many other pathogens such as HIV and *Mycobacterium tuberculosis*, which are also endemic in many areas endemic for schistosomiasis. Hence the development of central laboratories that are able to undertake molecular diagnostics targeting multiple infections in these regions would be a cost-effective approach for infection control, and rather than relying on less sensitive, less accurate diagnostics which miss infected individuals, thereby hindering control efforts. Other promising advances include the diagnostic application of the LAMP and RPA techniques, which are applicable to resource-poor settings. Simplifying available DNA extraction procedures, so that they are more convenientand less expensive, and the increased use of fingerprick blood spots and urine filtrates on filter papers [65], should be advocated for field-friendly DNA detection-based molecular diagnosis. Furthermore, improvement and adaption of direct PCR approaches, without the need for DNA extraction, modifications to nucleic acid amplification procedures making them simpler, reducing equipment requirements and expertise while maintaining accuracy, would invariably favor the field use of molecular assays. Altogether these advances have the potential to provide a mechanism for the wider application of more accurate and convenient DNA detection methods that will be invaluable in future schistosomiasis control and elimination efforts.

## Figures and Tables

**Table 1:** Advantages and limitations of different DNA diagnostics.

Assay Type	Advantages	Limitations	Relative Cost *	References
cPCR	- Low cost compared to qPCR and ddPCR - Can be multiplexed when target amplicon sizes are different	- Requires post PCR processing—running PCR products in a gel—which allows high chance of contamination - More time consuming and labor intensive compared to most other PCR methods - Prone to potential laboratory contamination with the need of manual handling of PCR products	$$	[42,43,44]
nPCR	- Use of two sets of primers improves the specificity while the need of two rounds of cPCR provide a higher sensitivity	- Relatively time consuming and complicated initial optimization process - Need to complete two rounds of cPCR amplifications, hence more time consuming and labor intensive - Prone to contamination with amplified PCR products	$$	[45,46]
qPCR	- Higher sensitivity compared to cPCR - Higher specificity, specifically when probes are used - No post PCR processing (such as running a gel in cPCR) required for final results - No manual handling of PCR products which limits the potential laboratory contamination - Can quantify the amount of amplicons (relative quantification) - Can be multiplexed for the detection of multiple species within a single reaction - Less time consuming and less labor intensive compared to cPCR	- Relatively time consuming and complicated initial optimization process in multiplexed assays - Requires triplicate reactions to improve the accuracy of final calculations - More expensive than cPCR, LAMP, direct PCR	$$$	[13,29,38]
LAMP	- Cost effective - Less equipment required - Can visualize the end products directly without running in a gel - Faster procedures compared to other types of PCR	- Relatively time consuming and complicated initial optimization process - Prone to carryover contamination	$	[47,48]
RPA	- Cost effective - Less equipment required - End products can be visualized on a chip/lateral flow device - Has a great potential to be developed as a point of care diagnostic tool	- Relatively time consuming and complicated initial optimization process - Prone to contamination with the need of transferring amplified products to a detection device	$	[49,50,51]
ddPCR	- Higher sensitivity compared to most other types of PCR - Higher specificity, specifically when probes are used - Can quantify the amount of amplicons (absolute quantification) - No manual handling of PCR products which limits the potential laboratory contamination - Can be multiplexed for the detection of multiple species within a single reaction	- Requires specific and expensive machinery for the initial establishment - Relatively time consuming and complicated initial optimization process in multiplexed assays	$$$	[35,52,53]
Direct PCR	- Raw sample is used as the template for PCR amplification, eliminating the need of a DNA extraction step - Less time consuming and convenient	- Initial development and optimization is relatively complicated and time consuming, as it is required to overcome PCR inhibitors in raw samples.	$	[54]

* The cost of diagnostics is given as a relative scale to each other: $—low, $$—moderate, $$$—high. The individual cost of DNA diagnostics can be variable depending on factors such as the type and brand of commercial reagents used and the regional source where these reagents are purchased. Abbreviations: cPCR: conventional PCR, ddPCR: droplet digital PCR, LAMP: Loop mediated isothermal amplification, nPCR: nested PCR, RPA: Recombinase polymerase amplification.

## Abbreviations

cfDNA	Cell-free DNA
CAA	Circulating anodic antigen
CCA	Circulating cathodic antigen
PCR	Polymerase chain reaction
cPCR	Conventional PCR
ddPCR	Droplet digital PCR
KK	Kato-Katz fecal smear
LAMP	Loop-mediated isothermal amplification
MDA	Mass drug administration
nPCR	Nested PCR
PCR-RFLP	Restriction fragment length polymorphism analysis of PCR products
POC	Point of care
qPCR	Real time quantitative PCR
RPA	Recombinase polymerase amplification
STH	Soil transmitted helminths
WASH	Water, sanitation and hygiene
